# Use of Relugolix for the Prevention of Impending Oliguria and Progressive Renal Failure in a Suspected Case of Prostate Carcinoma

**DOI:** 10.7759/cureus.77692

**Published:** 2025-01-20

**Authors:** Prem Kumar, Pranjal Prem, Ashish Raut, Shamim Ahmad, Smita Singh

**Affiliations:** 1 Urology, Ranchi Urology Centre, Ranchi, IND; 2 Obstetrics and Gynaecology/Urogynaecology, Ranchi Urology Centre, Ranchi, IND

**Keywords:** advanced prostate cancer, flare, oral lhrh antagonist, rapid and sustained testosterone suppression, relugolix

## Abstract

Androgen deprivation therapy for advanced prostate cancer has traditionally relied on luteinizing hormone-releasing hormone antagonists (LHRH). However, newer oral gonadotropin hormone-releasing hormone antagonists (GnRH) offer faster responses and fewer adverse effects. A 65-year-old male diabetic patient with a history of lower urinary tract symptoms and an indwelling Foley catheter for two weeks presented with respiratory difficulty, bilateral lower limb swelling, and decreased urine output. The investigation was suggestive of locally advanced prostate cancer with obstructive uropathy along with acute or chronic kidney disease. The patient was admitted to the ICU and stabilized. An urgent bedside prostate biopsy was performed. Relugolix 360 mg orally was given on the first day followed by 120 mg daily before histopathological confirmation due to impending oliguria and progressive kidney injury. Subsequent follow-up demonstrated clinical improvements, including reduced PSA and testosterone levels, confirming the efficacy of relugolix in managing advanced prostate cancer. Timely intervention and therapeutic adherence are crucial for optimal outcomes. Additionally, it highlights the preference for LHRH agonists in emergencies and the potential of oral GnRH antagonists like relugolix in prostate cancer management.

## Introduction

Prostate cancer is a significant global health concern that often exhibits no symptoms in its early stages. If left untreated, it can progress to serious complications such as obstructive uropathy due to an enlarged prostate. In this condition the flow of urine is blocked at the level of the bladder outlet or ureters, leading to back pressure changes in kidney and renal failure. Hormonal therapy plays a crucial role in managing prostate cancer because the growth of prostate cancer cells is fueled by testosterone, a male hormone. Prostate cancer, like breast cancer in females, is a hormone-dependent cancer, and hormonal manipulation remains the most important treatment. By reducing testosterone levels, hormonal therapy helps slow down or stop the progression of the disease. One common hormonal therapy involves the use of long-acting depot injections of luteinizing hormone-releasing hormone (LHRH) agonists. These drugs work by signaling the body to lower testosterone production via pituitary hormone. LHRH analogues are available in injectable form, with periodic injections ranging from one month to six months, which can lead to injection site complications, pain, and decreased compliance. However, they initially cause a temporary spike in testosterone levels, known as a “testosterone surge.” This surge can worsen symptoms such as urinary obstruction, bone pain, and, in rare cases, ureteral blockage or spinal cord compression [1, 2].

To mitigate these risks, most guidelines recommend co-administration of an androgen deprivation therapy (ADT) during the initial weeks of therapy [3, 4]. Despite their widespread use, LHRH agonists have limitations. They may not completely suppress another hormone follicle-stimulating hormone (FSH), which may act as a growth factor for prostate cancer cells by enhancing angiogenesis, cell proliferation, and apoptosis resistance, especially after ADT [5, 6]. To overcome this, a newer class of drugs called gonadotropin-releasing hormone (GnRH) antagonists has been introduced. These drugs, such as degarelix, directly and rapidly suppress both testosterone and FSH levels without causing an initial surge. However, degarelix's use is often limited by the need for monthly injections and associated injection-site reactions [7]. 

Relugolix, a novel oral GnRH receptor antagonist, offers a promising alternative. It rapidly inhibits the release of LHRH and FSH, effectively lowering testosterone levels. With a half-life of 25 hours and once-daily oral dosing, relugolix combines convenience with efficacy and safety, making it a promising alternative to traditional therapies for prostate cancer [8-10]. This report highlights the positive outcomes of relugolix in managing advanced prostate cancer and its potential advantages over traditional therapies.

## Case presentation

**Figure 1:**
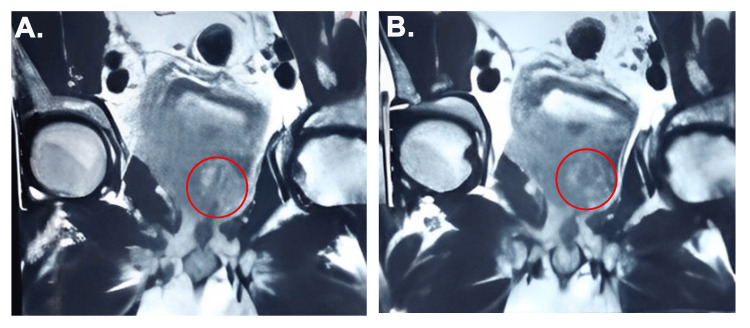
(A-B) Multiparametric MRI T2-weighted MRI reveals intermediate signal intensity suggestive of irregular mass in the prostate involving the left pelvic wall and abutting the wall of left external iliac vessels (red circle).

A 65-year-old diabetic male presented to the emergency department with respiratory difficulty, bilateral lower limb swelling, and decreased urinary output of approximately 650 ml over 24 hours. The patient had a history of lower urinary tract symptoms (LUTS) and chronic urinary retention, for which he had been catheterized two weeks earlier at another facility. Laboratory investigations revealed haemoglobin 9.0 gm%, elevated serum creatinine (5.45 mg/dL), and metabolic acidosis. Ultrasound imaging of the kidneys, ureters, and bladder (KUB) demonstrated bilateral moderate hydronephrosis, diffuse bladder wall thickening, and a grossly enlarged prostate. A digital rectal examination revealed a grade II hard prostate. A multiparametric MRI performed two weeks earlier showed a large lobulated lesion (PIRADS V), involving both sides of the prostate, extending to the bilateral seminal vesicles and posterior bladder wall, with metastatic involvement of multiple pelvic lymph nodes, findings consistent with locally advanced prostate cancer (Figure 1). His prostate-specific antigen (PSA) level was significantly elevated at 214 ng/mL. He was admitted to the intensive care unit (ICU) for respiratory support and renal monitoring in view of decreasing urinary output, rising serum creatinine, and fluid retention, for further management. His baseline creatinine value six months back was reported 1.9 mg/dl, suggestive of acute-on-chronic kidney disease due to obstructive uropathy. Nephrology consultation was sought, and the patient was started on intravenous antibiotics, furosemide infusion, soda bicarbonate infusion, fluid restriction, and BiPAP (bilevel positive airway pressure) support. Sugar monitoring was done and insulin was given accordingly.

**Figure 2:**
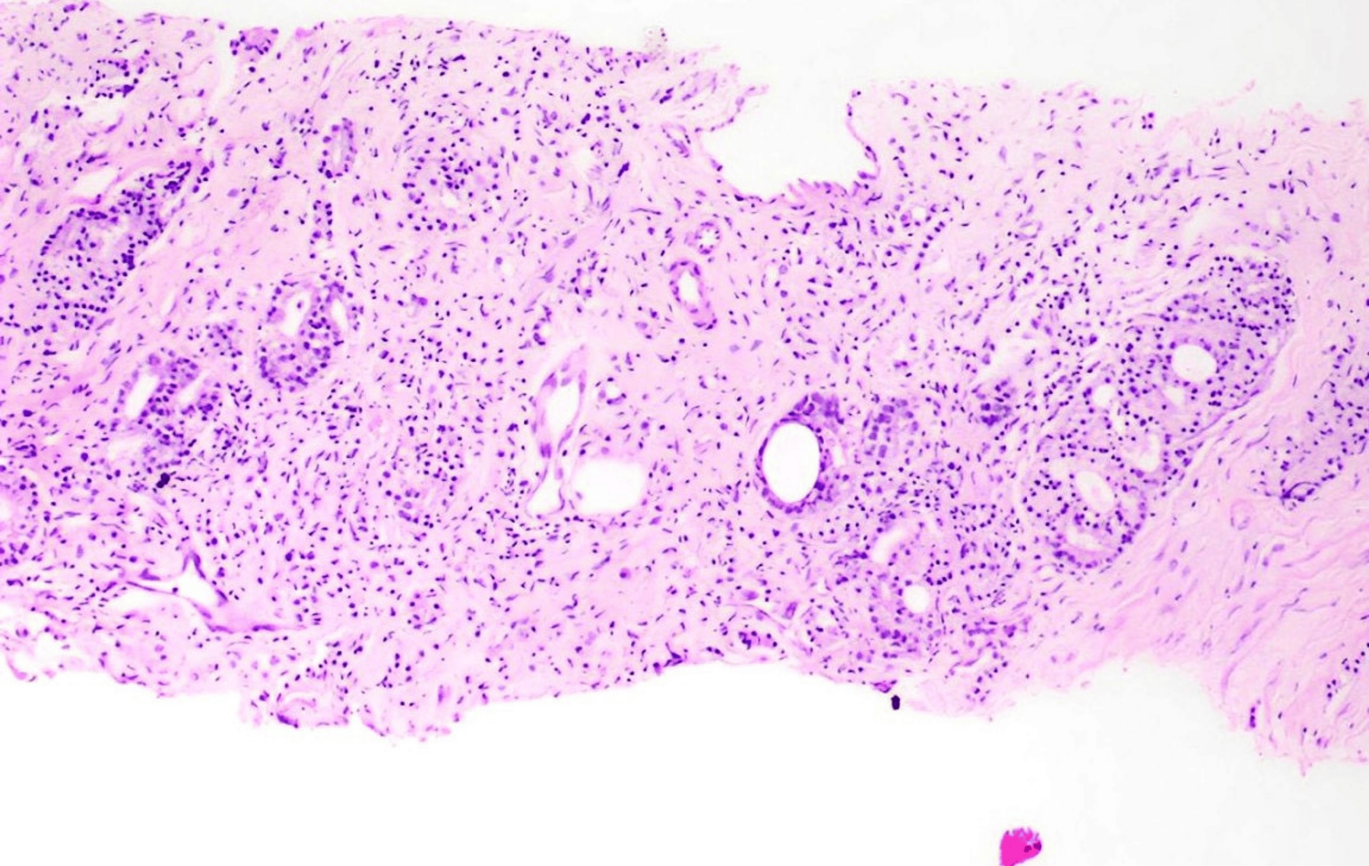
Histopathological examination Prostate core biopsy suggestive of prostatic (acinar) adenocarcinoma, Gleason score-4+3, histologic grade 3

An urgent transrectal prostatic biopsy was performed bedside, and the patient was started on relugolix (360 mg once on the first day, followed by 120 mg once daily) before histopathological confirmation due to impending oliguria and acute kidney injury with obstructive uropathy. Histopathological examination confirmed prostatic adenocarcinoma with a Gleason score of 4+3=7, involving 60% of the core, HG3 (High grade 3), without evidence of perineural or lymphovascular invasion (Figure 2).

**Figure 3:**
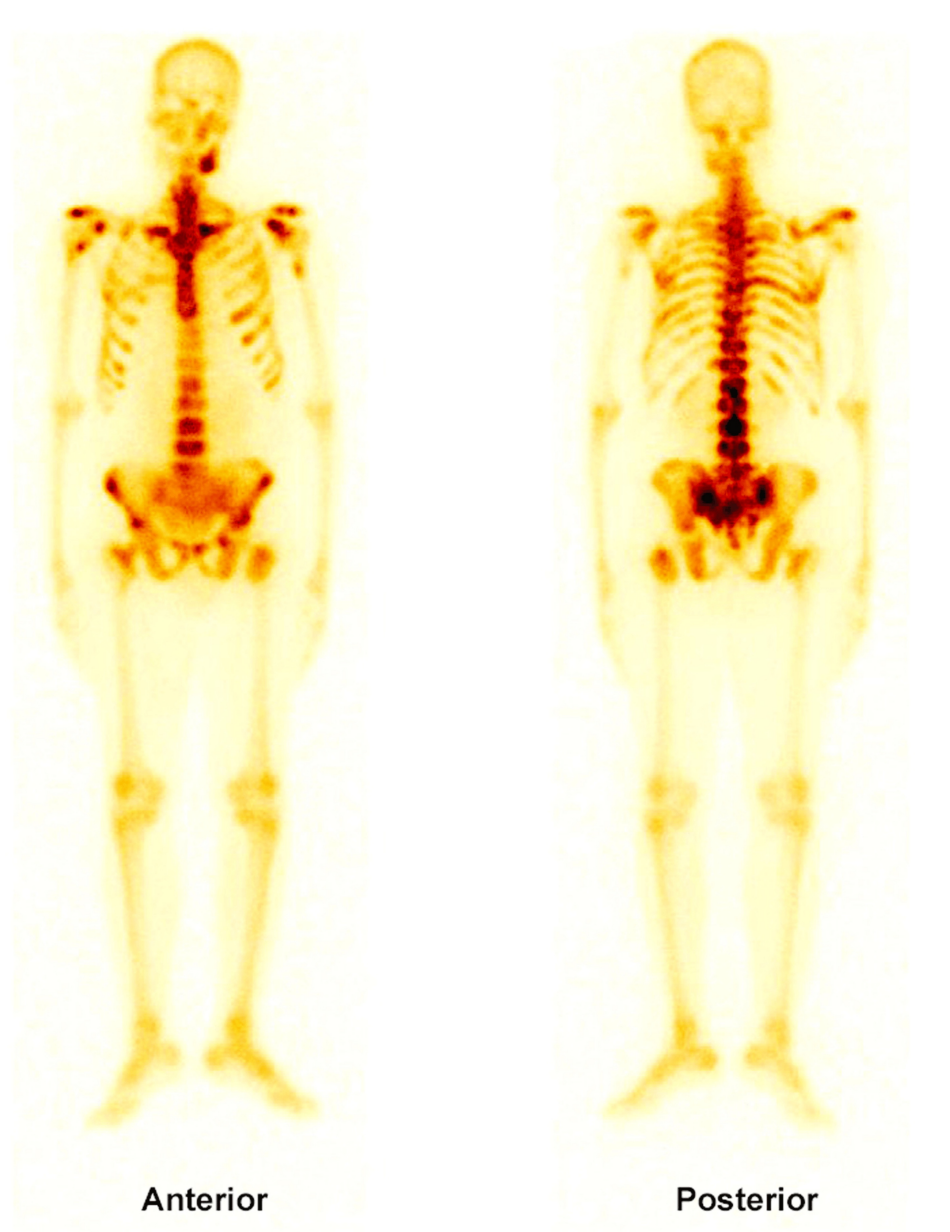
Bone scan image Bone scan imaging unveils extensive skeletal metastasis in patient

The patient showed gradual improvement in urinary output and a gradual decline in serum creatinine with symptomatic improvement, indicating relief from urinary obstruction and improvement in renal function. Subsequent bone scans revealed multiple skeletal metastases (Figure 3).

Subsequent blood parameters measured after 12 days showed a significant reduction in serum testosterone and PSA levels. At three weeks, the patient demonstrated marked clinical improvement, with PSA levels decreasing from 214 ng/mL to 6.53 ng/mL and testosterone levels falling to below 10 ng/dL (Table 1).

**Table 1:** Patient clinical outcome The biochemical parameters of the patient demonstrates a positive response to relugolix treatment, which was started on Day 0. Reference range: prostate-specific antigen, 0-4 ng/mL; creatinine, 0.7-1.3 mg/dL; testosterone, 280-1,100 ng/dL *Serum testosterone test is done only to look for the response of hormonal treatment and it is not required to start treatment.

Visit	Prostate-specific antigen (ng/mL)	Creatinine (mg/dl)	Testosterone (ng/dL)
Baseline (Day 0)	214	5.45	Not available*
Follow-up 1 (12 days)	54.39	3.35	16.16
Follow-up 2 (22 days)	6.53	2.32	<10
Follow-up 3 (44 days)	3.2	3.6	<10

**Figure 4:**
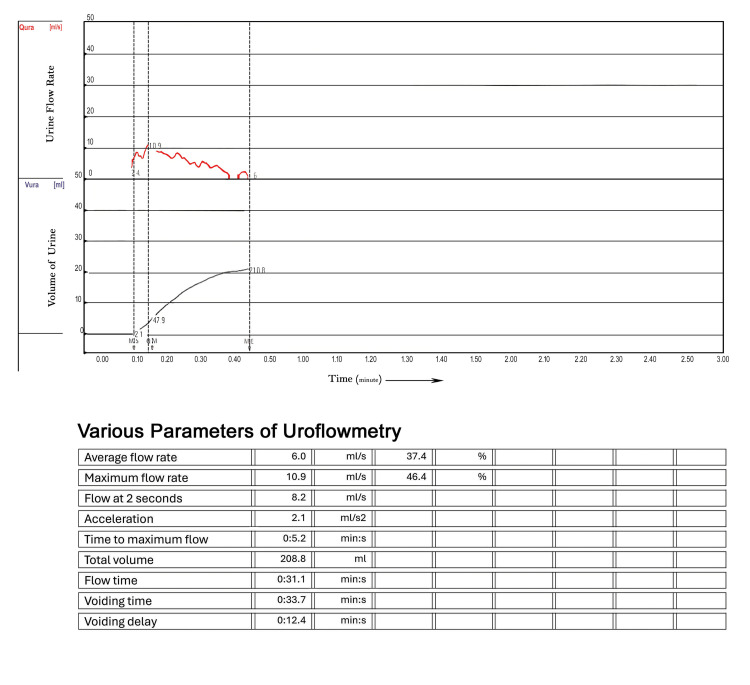
Follow-up uroflowmetry report Uroflowmetry: three weeks post-treatment following voiding trial (Vmax: 10.9 ml/s, Vavg: 6.0 ml/s), suggestive of treatment efficacy *Red curve: flow rate of urine; black curve: volume of urine; min s- minutes and seconds

The Foley catheter was removed, and the patient was able to void with reasonable flow and minimal obstruction. His LUTS were significantly relieved. Uroflowmetry was performed, showing a maximum flow rate (Vmax) of 10.9 mL/s and an average flow rate (Vavg) of 6.0 mL/s (Figure 4).

The patient continued on relugolix and experienced sustained symptomatic and clinical improvements. At the six-week follow-up, persistent hydronephrosis was noted, for which bilateral retrograde pyelography (RGP) and stenting were done. Despite these interventions, renal function did not improve significantly, suggesting chronic kidney disease and no significant residual obstruction of ureters. The absence of residual obstruction supports the fact that relugolix effectively alleviated ureteric obstruction.

## Discussion

Since the identification of the GnRH pathway by Schally et al. in 1971 [5], LHRH agonists emerged as the primary therapy for advanced prostate cancer in men. Despite challenges in creating safe and efficient GnRH antagonists, the development of LHRH agonists ensued as an alternative [11, 12]. Long-term administration of LHRH agonists desensitizes the pituitary receptor, suppressing luteinizing hormone and testosterone production, thereby blocking pulsatile GnRH secretion. Initially, and to a lesser extent with subsequent doses, LHRH agonists cause a surge in luteinizing hormone, FSH, and testosterone, with delayed testosterone suppression. The clinical significance of this acute testosterone surge remains under debate, as does the efficacy of anti-androgen pre-treatment [13, 14].

Moreover, extended use of LHRH agonists does not effectively inhibit FSH, the importance of which remains uncertain. Additionally, the potential activation of extra pituitary receptors by LHRH agonists, such as those expressed in the heart, prostate, and bladder, is a relatively unexplored area [11,15]. Injectable peptide and oral non-peptide antagonists rapidly and directly inhibit the production of luteinizing hormone, FSH, and testosterone. However, safety considerations, including potential reactions at injection sites and hypersensitivity, have constrained their utilization [12,16]. The oral GnRH antagonist, relugolix, rapidly reduced testosterone to levels considered castrate by the fourth day. Conversely, in the leuprolide group, mean testosterone levels initially spiked very high before declining to castrate levels by Day 29. Treatment with relugolix not only eliminates the risks associated with testosterone surges and the necessity for antiandrogen therapy to prevent symptom flares but also offers the advantage of promptly suppressing testosterone. This rapid reduction may prove advantageous for both clinicians and patients contemplating further antineoplastic interventions like radiation or chemotherapy, positioning it as a promising oral alternative to injectable androgen deprivation therapies for prostate cancer [17,18].

In a predetermined safety evaluation, the relugolix group exhibited a reduced occurrence of significant adverse cardiovascular events compared to the leuprolide group, although there was a higher frequency of mild to moderate diarrhea. Following 48 weeks of therapy, the relugolix group experienced a 54% decrease in the risk of major adverse cardiovascular events compared to the leuprolide group. Subsequent subgroup examination indicates that this contrast might have been more pronounced among individuals with pre-existing cardiovascular risk factors [19]. Injectable LHRH antagonists require monthly depot injections administered by healthcare professionals, often necessitating clinic visits. In contrast, relugolix can be administered orally, providing patients with the convenience of self-administration at home. This eliminates the need for frequent clinic visits and reduces the burden associated with injections, potentially improving treatment adherence and patient satisfaction [20].

The cost-effectiveness and accessibility of treatment are important considerations in healthcare decision-making. Injectable LHRH antagonists may incur higher costs due to the need for healthcare professional administration and frequent clinic visits. Relugolix, as an oral medication, may offer cost advantages and improved accessibility, particularly in settings where healthcare infrastructure for injection administration is limited.

Patient preference and quality of life are critical factors in treatment decision-making. The convenience of oral administration with relugolix may be preferred by some patients, particularly those averse to injections or with logistical challenges accessing healthcare facilities. Improving quality of life by reducing treatment-related burdens and enhancing patient autonomy is a significant advantage of relugolix. In this case, considering persistent bilateral hydronephrosis and raised serum creatinine even after relugolix treatment, bilateral stenting and bilateral RGP were carried out. Despite relieving the obstruction of the system, the patient’s renal function did not improve significantly, suggesting CKD. Consequently, relugolix shows promise as an effective treatment for deobstructing the system. It may be particularly beneficial in managing impending ureteric obstruction oliguria and impending urinary retention in the setting of advanced prostate cancer.

LHRH antagonists are preferred in emergency situations such as impending urinary retention, impending ureteric obstruction, or impending paraplegia caused by spinal cord compression. The "flare" effect associated with LHRH agonists can exacerbate these conditions, potentially leading to acute urinary retention, anuria, or paraplegia. Therefore, LHRH agonists should be avoided in such scenarios. In cases where the diagnosis is uncertain, using long-acting injectable LHRH agonists is not ideal, as their prolonged effects may complicate management. Oral LHRH antagonists, with their shorter half-life, offer a distinct advantage; they can be discontinued promptly if the diagnosis is not confirmed, minimizing long-term side effects. Moreover, LHRH antagonists are suitable for hormonal ablation in both emergency and non-emergency settings, including locally advanced and metastatic prostate cancer.

## Conclusions

In conclusion, both injectable LHRH antagonists and oral relugolix are effective options in the management of prostate cancer. While injectable formulations have an established role in clinical practice, oral GnRH antagonists like relugolix offer additional benefits, including greater convenience, improved safety profiles, and enhanced patient satisfaction. However, further research and real-world evidence are essential to fully compare the long-term outcomes and effectiveness of these treatment modalities. Such data will support personalized and evidence-based decision-making in prostate cancer care.
